# An Orphan CpG Island Drives Expression of a *let-7* miRNA Precursor with an Important Role in Mouse Development

**DOI:** 10.3390/epigenomes3010007

**Published:** 2019-03-13

**Authors:** Martha V. Koerner, Kashyap Chhatbar, Shaun Webb, Justyna Cholewa-Waclaw, Jim Selfridge, Dina De Sousa, Bill Skarnes, Barry Rosen, Mark Thomas, Joanna Bottomley, Ramiro Ramirez-Solis, Christopher Lelliott, David J. Adams, Adrian Bird

**Affiliations:** 1 Wellcome Centre for Cell Biology, University of Edinburgh, Michael Swann Building, Max Born Crescent, Mayfield Road, Edinburgh EH9 3BF, UK; 2 Wellcome Trust Sanger Institute, Hinxton, Cambridge CB10 1SA, UK

**Keywords:** micro-RNA, Let-7, mouse genetics

## Abstract

Most human genes are associated with promoters embedded in non-methylated, G + C-rich CpG islands (CGIs). Not all CGIs are found at annotated promoters, however, raising the possibility that many serve as promoters for transcripts that do not code for proteins. To test this hypothesis, we searched for novel transcripts in embryonic stem cells (ESCs) that originate within orphan CGIs. Among several candidates, we detected a transcript that included three members of the *let-7* micro-RNA family: *Let-7a-1*, *let-7f-1*, and *let-7d*. Deletion of the CGI prevented expression of the precursor RNA and depleted the included miRNAs. Mice homozygous for this mutation were sub-viable and showed growth and other defects. The results suggest that despite the identity of their seed sequences, members of the *let-7* miRNA family exert distinct functions that cannot be complemented by other members.

## 1. Introduction

CpG islands (CGIs) are domains of approximately 1000 base pairs that include the promoters of most mammalian genes [1]. Within CGIs, the dinucleotide sequence is about 10 times more frequent than in the bulk genome and usually lacks DNA methylation. It was shown previously that the genomes of mice and humans contain similar numbers of CGIs, but many are not associated with annotated transcripts. We hypothesised that many of these so-called “orphan CGIs” are promoters for non-coding transcripts. Given the importance of the expression of non-coding RNAs for gene regulation in development, as elegantly dissected by the laboratory of Denise Barlow [2,3,4,5], we set out to define novel transcripts driven by CGI promoters. In this study, we investigated a transcript that serves as a precursor for *let-7* micro-RNAs. Members of the *let-7* family of (*let-7a*/*b*/*c*/*d*/*e*/*f*/*g*/*i* and *miR-98*) share a common seed sequence, nucleotides 2 through 8 of their 5′ ends, which is required for target recognition [6]. They were originally discovered in *Caenorhabditis elegans*, where they play crucial roles in the temporal regulation of development, with decreased expression resulting in over-proliferation and the absence of terminal differentiation. Not only is *let-7* highly conserved throughout the animal kingdom, but its role in normal differentiation and development is also relatively consistent, a common theme being the consolidation of gene expression programmes in differentiated cells. In mouse embryonic stem cells (ESCs), for example, mature *let-7* is undetectable, but accumulates during differentiation [7,8]. This scenario notionally fits with the frequent absence of *let-7* miRNA in cancer cells, which have resumed a proliferative state, and with the finding that elevated *let-7* expression can block tumour formation, progression, and metastasis [9]. While previous studies have established the *let-7* expression pattern during vertebrate development, details of its direct involvement in developmental processes have been difficult to establish [8,10,11]. Different *let-7* family members are considered likely to have highly redundant roles, which might necessitate knocking out all 13 members simultaneously to study the function. Here, however, we show that loss of a single precursor RNA overlapping three *let-7* family members severely reduces mouse viability coincident with increased body length, weight, and other phenotypes. These results suggest that these members of the *let-7* family exert non-redundant functions, which the availability of this mouse model promises to elucidate.

## 2. Results

### 2.1. An Orphan CpG Island Serves as a Promoter for a Conserved miRNA Precursor

**Figure 1:**
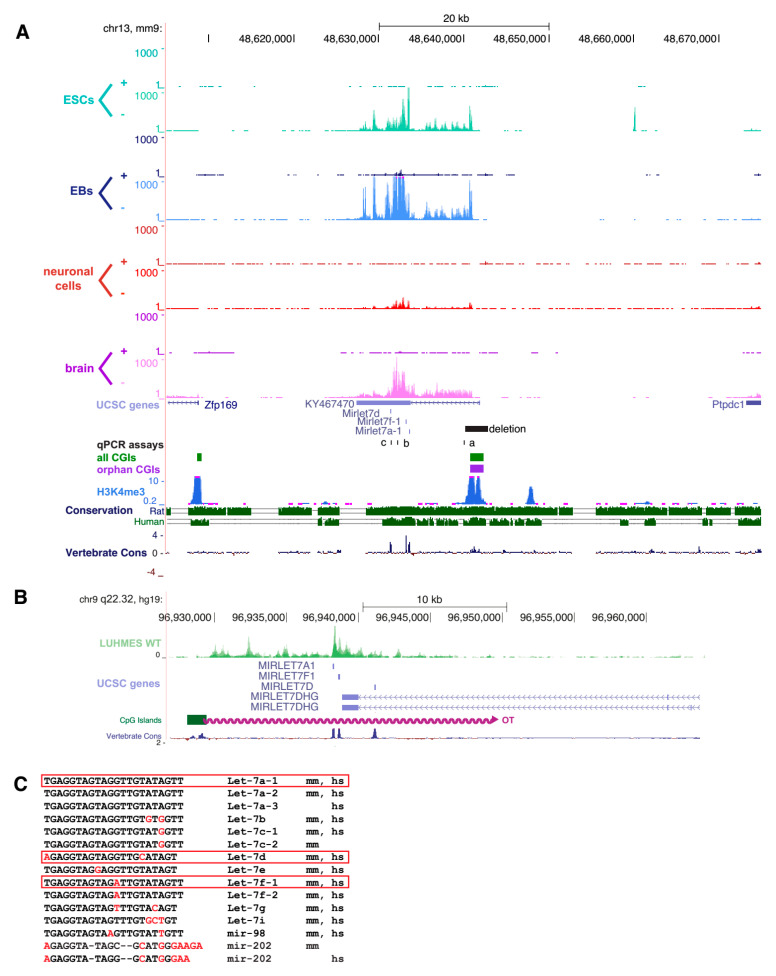
A conserved long ncRNA overlapping three miRNAs is expressed from an orphan CpG island. (**A**) Mouse RNA-Seq data displayed on the UCSC genome browser on mouse chromosome 13 (mm9). RNA-Seq reads mapping the + and - strand are shown separately for RNA harvested from embryonic stem cells (ESCs), embryoid bodies (EBs), neuronal cells, and adult mouse brain. CpG islands were annotated according to [12] and grouped into “all CGIs” (green bars) and “orphan CGIs” (purple bars). During the course of the study, a transcript originating from this orphan CGI was annotated: *KY467470* as a transcript overlapping three annotated miRNAs. The region which was deleted in mice is shown as a black bar. Primers for qPCR assays (a, b, c) are shown as black lines. H3K4me3 and conservation tracks from the UCSC genome browser are shown. (**B**) Human RNA-Seq data displayed on the UCSC genome browser on human chromosome 9 (hg19). RNA-Seq data from human wildtype LUMES cells are plotted in green. This transcript starts from a CGI syntenic to the mouse orphan CGI in A) (green bar) and overlaps the same three miRNAs. (**C**) Sequences of the *let-7* family. Bases differing from *let-7a* are shown in red, missing bases as -. Species of origin of the miRNA is indicated as mice (mm) and/or human (hs). miRNAs framed in red are those shown in (**A**) and (**B**).

We performed RNA-Seq to identify transcripts originating from orphan CpG islands (CGIs) that were identified previously [12]. RNA was derived from three stages of differentiation: Proliferating E14Tg2a embryonic stem cells prior to differentiation; embryoid bodies (EBs) representing an intermediate stage of differentiation; and derivatives that had been further differentiated into neuronal cells. For comparative purposes, we also analysed adult mouse brains from wildtype male C57BL/6JCrl mice. Extracted RNA was subject to strand-specific RNA-Seq and reads were plotted onto the University of California Santa Cruz (UCSC) genome browser. By visual inspection, we identified a long transcript, which appeared to originate from CGI-5563 located on chromosome 13 (mm9, chr13:48640767-48642340) (Figure 1A), which was recently annotated as *KY467470*. We found that *KY467470* is expressed in all tissues, with neuronal cells showing the lowest read density, and embryoid bodies the highest. *KY467470* overlapped three annotated members of the *let-7* miRNA family: *Let-7a-1*, *let-7f-1*, and *let-7d*. The CGI as well as the three miRNAs showed significant conservation in mammals, including humans, where the RNA-Seq of neuronal cells reveals a long transcript overlapping the same three miRNAs (see Figure 1B). We conclude that *KY467470* represents a precursor RNA from which the mature *let-7* RNAs are processed.

**Figure 2:**
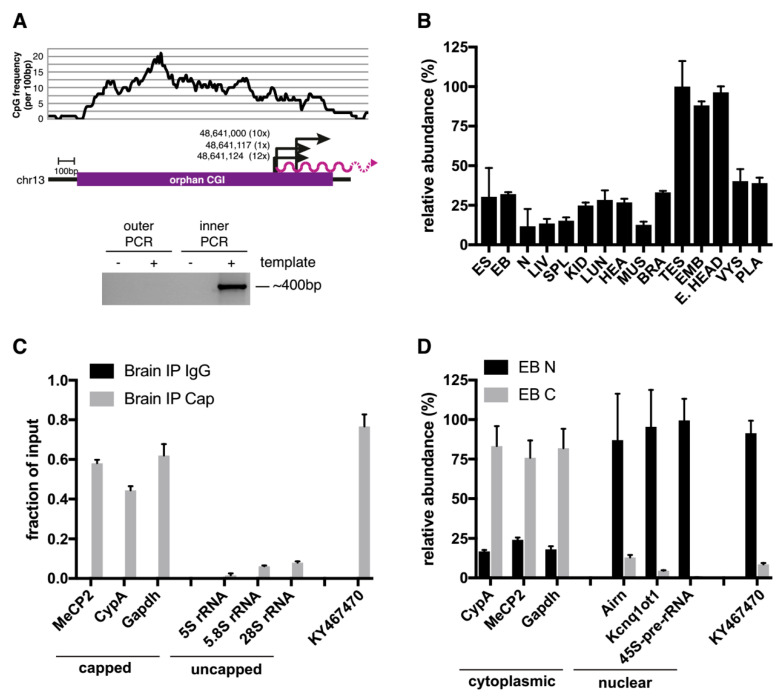
KY467470 originates from the orphan CGI, shows widespread expression and is capped and nuclear localised. (**A**) 5′ RACE maps the transcription start site (TSS) of *KY467470*. A plot of the CpG frequency is shown on the top. Below, a purple bar indicates the orphan CGI as mapped by [12,14]. Arrows indicate the three TSSs identified by sequencing of the 5′ RACE products with bp coordinates shown next to the arrows. Numbers in brackets indicate the number of sequence reads for each TSS. The purple wavy line indicates *KY467470*. The photograph of the gel below shows the products of the outer and inner PCRs according to the 5′ RACE procedure. (**B**) qPCR analysis measuring the relative abundance of *KY467470* in different mouse cells/tissues. The location of the primers is shown in Figure 1A). Tissues are denoted as follows: Embryonic stem cells (ES); embryoid bodies (EB); neuronal cells (N); liver (LIV); spleen (SPL); kidney (KID); lung (LUN); heart (HEA); thigh muscle (MUS); brain (BRA); testis (TES); 12.5 dpc embryonic trunk (EMB); 12.5 dpc embryonic head (E. HEAD); visceral yolk sac (VYS); placenta (PLA). Shown are mean and standard deviation for two (ES, EB, N) or three (all other tissues) biological replicates. (**C**) qPCR analysis of 5′-Cap IP. Mock IP is shown in black, Cap-IP in grey. MeCP2, *Cyclophilin A* (*CypA*), and *Gapdh* were controls for capped mRNAs, whereas 5S rRNA, 5.8S rRNA, and 28S rRNA were controls for uncapped RNAs. (**D**) qPCR analysis of relative transcript abundance in the nuclear (N; black) or cytoplasmatic (**C**; grey) fraction in embryoid bodies (EB). Controls for cytoplasmic RNAs were *CyclophilinA* (CypA), *Gapdh*, and controls for nuclear localised RNAs were *Airn*, *Kcnq1ot1*, and the *45S-pre-rRNA*.

Using 5′ RACE, we identified three transcription start sites (TSSs) of *KY467470*, all located within the last third of the CGI (Figure 2A). Of the 23 sequenced clones, 12 originated from TSS1 (chr13:48,641,124), 1 from TSS2 (chr13:48,641,117), and 10 from TSS3 (chr13:48,641,000). Transcript abundance was determined by qPCR analysis in male adult mouse tissues (liver, spleen, kidney, lung, heart, thigh muscle, brain, testis) and embryonic and extra-embryonic mouse tissues (embryonic trunk, embryonic head, visceral yolk sac, placenta). This revealed widespread expression of *KY467470*, most prominently in the testis, embryonic trunk, and embryonic head (see Figure 2B). We used publicly available DNA methylation data to determine the methylation status of this CGI in different tissues. In agreement with the widespread expression of the transcript, this CGI is unmethylated in all tissues analysed [13]. To see if *KY467470* was capped, we performed immunprecipitation (IP) using an antibody against the 5′ cap of mRNAs. Using known capped mRNAs (*MeCP2*, *CyclophilinA*, *Gapdh*) and uncapped rRNAs (*5S rRNA*, *5.8S rRNA*, and *28S rRNA)* as controls, the results demonstrated that *KY467470* is capped (see Figure 2C). We next asked whether *KY467470* was nuclear or cytoplasmic using qPCR on fractionated cells. Known mature mRNAs (*Mecp2*, *CyplophilinA*, and *Gapdh*) were located in the cytoplasm, whereas known nuclear RNAs (*Airn*, *Kcnq1ot1*, and the *45S-pre-rRNA*) were located in the nuclear fraction. *KY467470* showed clear enrichment in the nuclear fraction, indicating that most are not exported to the cytoplasm (see Figure 2D).

### 2.2. A Knock-Out (KO) of the Orphan CGI in Mice Abrogates Expression of let-7 Subtypes

**Figure 3:**
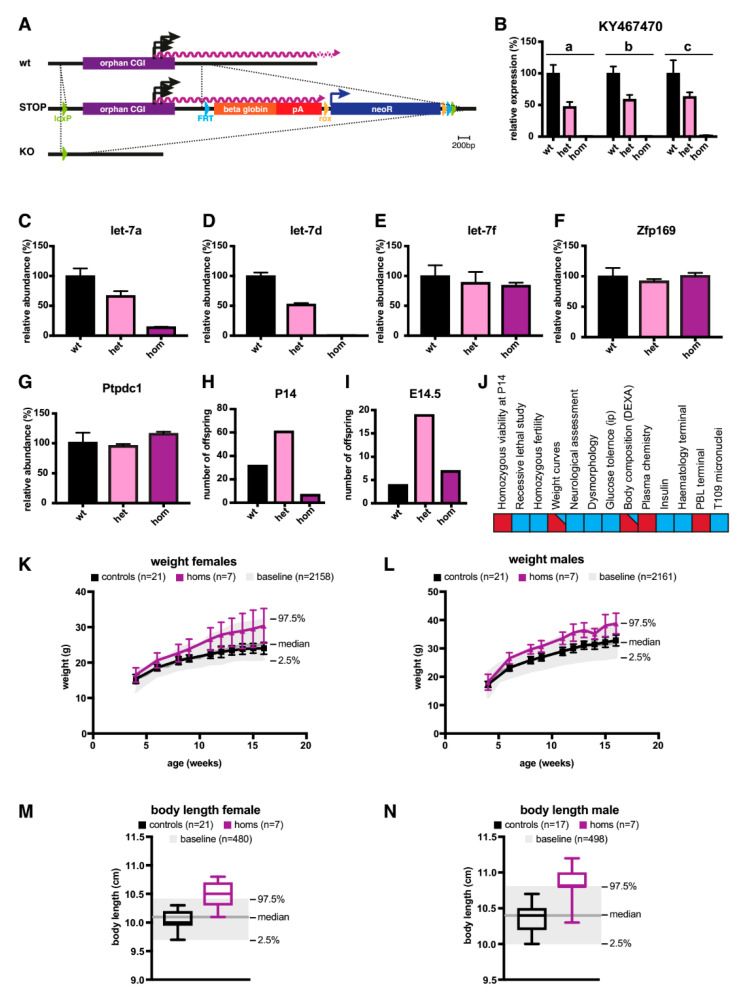
Knock-out of the orphan CGI leads to sub-viability, increased weight, and increased body length. (**A**) Scheme showing the strategy to knock-out the orphan CGI. At the wildtype locus, the orphan CGI (purple bar) drives expression of *KY467470* (purple wavy line). A loxP site was integrated in the 5′ of the orphan CGI. Downstream of the CGI, a STOP/selection cassette containing a flippase recognition target (FRT) site, a beta-globin stop cassette, a rox site, a neomycin-resistance cassette, a second rox, a second FRT, and finally a second loxP site was inserted to create a STOP allele. The STOP allele was converted into a KO allele by CRE recombination, deleting the orphan CGI as well as the STOP/selection cassette. (**B**) qPCR analysis was used to detect the relative abundance of *KY467470* in wildtype, heterozygous, and homozygous KO animals. Shown are three different assays along the length of KY467470 (a, b, c; for location see Figure 1A). The mean and standard deviation of three animals/genotype are shown. Levels were normalised to *CyclophilinA*. (**C**) qPCR analysis was used to detect the relative abundance of the *let-7a* mature miRNA in wildtype, heterozygous, and homozygous KO animals. The mean and standard deviation of three animals/genotype are shown. Levels were normalised to *Snord68*. (**D**) qPCR analysis was used to detect the relative abundance of the *let-7d* mature miRNA in wildtype, heterozygous, and homozygous KO animals. The mean and standard deviation of three animals/genotype are shown. Levels were normalised to *Snord68*. (**E**) qPCR analysis was used to detect the relative abundance of the *let-7f* mature miRNA in wildtype, heterozygous, and homozygous KO animals. The mean and standard deviation of three animals/genotype are shown. Levels were normalised to *Snord68*. (**F**) qPCR analysis was used to detect the relative abundance of *Zfp169*. Details as in (**B**). (**G**) qPCR analysis was used to detect the relative abundance of *Ptpdc1*. Details as in (**B**). (**H**) The number of offspring at P14 being wildtype, heterozygous, or homozygous for the KO allele. Chi squared = 17.340 (2 degrees of freedom), 2-tailed *p* value = 0.0002. (**I**) The number of offspring at E14.5 that are wildtype, heterozygous, or homozygous for the KO allele. Chi squared = 2.733 (2 degrees of freedom), 2-tailed *p* value = 0.2550. (**J**) Scheme indicating the detection of phenotypes by measuring various parameters. A blue square indicates the absence of a detectable difference from the wildtype, a red square indicates an automatically detected phenotype, and a red square with a blue triangle indicates that the automatic call was not significantly different from the wildtype, but this was manually overridden based on observation, resulting in a significant phenotype, comparing mutant data to a reference range generated from age, sex, and strain-matched wildtypes. (**K**) Body weight in females homozygous for the KO allele (purple), or controls (black). Shown are the mean and standard deviation. Baseline values (median, 97.5% and 2.5%) are shown in grey. Here, a manual call for a significant phenotype was made, which was supported by observations in males. (**L**) Body weight in males. A manual call was based on the appearance of a phenotype that, although observed at a low frequency in the mutant, is rarely observed in the baseline wildtype population. (**M**) Body length in females. The box extends from the 25th to the 75th percentile, the horizontal line is the median, and whiskers denote the minimum and maximum values. Here, a manual call for a significant phenotype was made, which was supported by an observation in males. (**N**) Body length in males. A manual call for significance was based on the appearance of a phenotype that, although observed at a low frequency in the mutant, is rarely observed in the baseline wildtype population.

To confirm that the CGI indeed served as the promoter for *KY467470*, and to identify the biological consequences of its abrogation in mice, we genetically modified the CGI in C57BL/6N ESCs. We inserted loxP sites upstream and downstream of the CGI. The knock-in cassette also contained elements that were not utilised in this study (see Materials and Methods). Chimeras were generated by blastocyst injection and the CGI cassette flanked by loxP sites was deleted using cell-permeable CRE recombinase (see Figure 1A and Figure 3A). After confirming successful deletion, we analysed *KY467470* expression in the adult mouse brain. The relative abundance of *KY467470* in heterozygous knock-out (KO) mice approximately 1 kb downstream from the TSS was reduced to 47%, and in homozygous KO mice to 0.3%. Quantitative PCR assays using primers located further downstream confirmed drastic depletion of *KY467470* by deletion of its CGI promoter (see Figure 3B).

The *let-7* family of miRNAs comprises 13 members in mice and humans, but some are specific to each species, whereas others are conserved in both species (see Figure 1C). Of the three encoded in *KY467470*, *let-7a* and *let-7f* are each present at two loci in mice, whereas *let-7d* is only found once in the genome, at the locus studied here. In the brain, we found that *let-7a* was reduced to 67% in heterozygous KO mice, and 15% in homozygous KO mice (see Figure 3C). This indicates that the majority of mature *let-7a* in the mouse brain originates from our targeted *let-7a-1* locus, with only a minority derived from the second locus, *let-7a-2* on chromosome 9. The relative abundance of *let-7f* in the brain was minimally affected by abrogation of *KY467470*, as heterozygous KO mice expressed 89% and homozygous KO mice expressed 84% of wildtype levels (see Figure 3E). We conclude that *let-7f-2* on chromosome X is the main contributor to mature *let-7f* levels in the mouse brain. In contrast, *let-7d* was most dramatically affected by the mutation, as it is exclusively encoded at this locus in the mouse genome. The abundance of *let-7d* was reduced by half (53%) in heterozygous mice and it was essentially absent (0.4%) in homozygotes (see Figure 3D). To test if the deletion of the CGI or loss of associated transcripts had an effect on the two flanking genes, *Zfp169* and *Ptpdc1*, we analysed brains from wildtype, heterozygous, and homozygous KO animals. Both genes showed the same mRNA abundance in all tested genotypes, indicating that neither the deleted CGI, *KY467470*, nor depletion of the associated miRNAs influence the expression of these genes (Figure 3F,G).

Analysis at postnatal day 14 (P14) of the progeny from a cross involving heterozygous male and female mice showed that homozygous animals were present at a reduced frequency. Out of 100 offspring, only 7 were homozygous for the KO allele compared with the expectation of 25 (see Figure 3H). When we analysed the genotype distribution at E14.5, homozygous KO mice were present at a Mendelian ratio with no obvious phenotype (see Figure 3I). We conclude that the deletion of the CGI and associated loss of *KY467470* confers a subviable phenotype, affecting animals between E14.5 and P14. Available homozygous KO mice were then subjected to a panel of phenotypic assays (see Figure 3J). Both males and females showed increased body weight (see Figure 3K,L) during adulthood and this correlated with an increased body length in both sexes (see Figure 3M,N).

**Figure 4:**
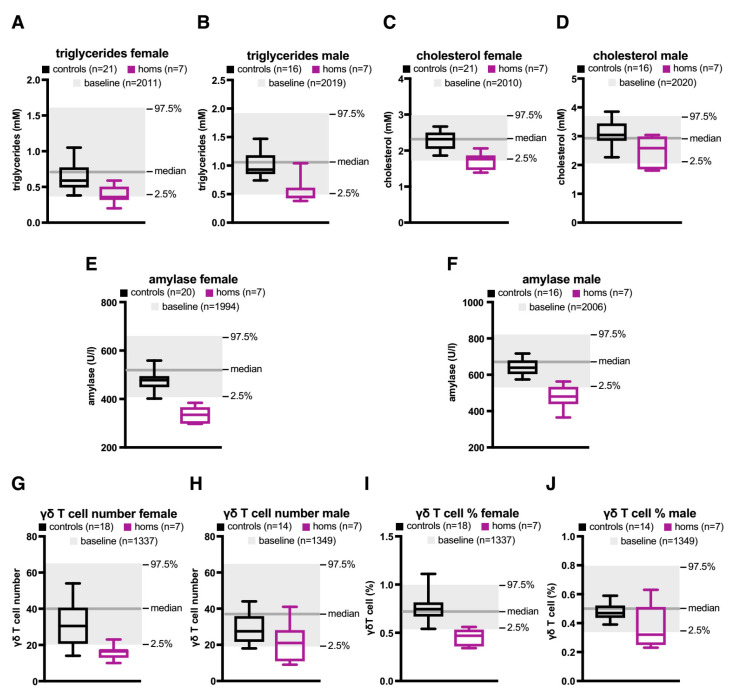
Knock-out of the orphan CGI affects plasma chemistry and gamma delta T-cells. (**A**) Triglyceride levels in females homozygous for the KO allele (purple), or controls (black). The box extends from the 25th to the 75th percentile, the horizontal line is the median, and whiskers denote the minimum and maximum values. Baseline values (median, 97.5% and 2.5%) are shown in grey. Automatic significance called. (**B**) Triglyceride levels in males. Automatic significance called. (**C**) Cholesterol levels in females. Manual call for significance is supported by an observation in another parameter (triglycerides). Manual call is based on mutant data being clustered across the periphery and outside the boundaries of the reference range. The auto-call rule is considered too stringent on this occasion. (**D**) Cholesterol levels in males. No significant difference. (**E**) Amylase levels in females. Automatic significance called. (**F**) Amylase levels in males. Automatic significance called. (**G**) Gamma delta T cell number in females. Automatic significance called. (**H**) Gamma delta T cell number in males. No significant difference. (**I**) Gamma delta T cell % in females. Manual call is supported by an observation in another parameter (gamma delta T cell number). (**J**) Gamma delta T cell % in males. No significant difference.

Examination of plasma revealed further abnormalities, including reduced triglyceride levels in males and females (see Figure 4A,B) and reduced cholesterol levels in females (see Figure 4C). Note that although cholesterol levels in homozygous KO males were also lower than in controls, the difference did not reach statistical significance (see Figure 4D). Plasma amylase levels were significantly reduced in both male and female homozygous KO mice (see Figure 4E,F). Whereas most immune cells were not affected by the KO, measurement of the number and fraction of gamma delta T-cells indicated another sex-specific difference. Whereas homozygous KO females showed a significantly reduced number and percentage of gamma delta T-cells, there was no statistically significant difference in males (see Figure 4G–J).

The phenotypes described here were identified as significant using the reference range method (see Materials and Methods for details). A comprehensive list of tested phenotypes is also available on the International Mouse Phenotyping Consortium (IMPC) website (http://www.mousephenotype.org/data/genes/MGI:5670665). Note that the IMPC uses different methods to identify significance and as a result more phenotypes are listed there than using our more conservative calling method (see Materials and Methods for details). Additional phenotypes listed on the webpage of the IMPC that did not reach significance using our approach were decreases in circulating total protein, glycerol, low density lipoprotein (LDL) cholesterol, high density lipoprotein (HDL) cholesterol, free fatty acid, glucose, fasted glucose (males only), blood urea nitrogen, as well as increases in magnesium and chloride. Additional effects observed in the immune system were decreases in the numbers of T-cell subtypes, alpha-beta T-cells, CD4-positive alpha beta T-cells, CD8-positive alpha beta T-cells, and effector memory CD4-positive alpha-beta T-cells. Furthermore, an increased bone mineral content was detected.

## 3. Discussion

The broad goal of this study was to identify and characterise non-coding RNAs that originate from orphan CGIs. We were motivated by the pioneering work on regulatory non-coding RNAs by Barlow and colleagues who showed that the *Airn* transcript regulates overlapping and non-overlapping genes and is expressed from a CGI promoter [3,4,5,15,16]. We identified an orphan CGI as the promoter driving the precursor transcript for three miRNAs. MicroRNAs are a class of small regulatory RNAs that play a role in most cellular processes. Primary precursor transcripts are first cleaved in the nucleus to give hairpin precursors (pre-miRNAs) and are then exported to the cytoplasm [17,18], where they are further processed by DICER [19,20,21,22]. In agreement with this scenario, we find that the miRNA precursor, KY467470, is retained in the nucleus. The resulting mature miRNAs are incorporated into the RNA-induced silencing complex, where they suppress target mRNAs via complementary base pairing [23]. In mammals, the *let-7* family consists of 13 members all of which contain an identical seed sequence, which is the major determinant of target selection. Targets include the oncogenes, *RAS* [24], *HMGA2* [25,26], and *c-MYC* [27], and multiple genes involved in pluripotency maintenance [28]. While levels of precursor RNAs are comparable between undifferentiated and differentiated cells, mature *let-7* is detected only after differentiation of ESCs [7], indicating that post-transcriptional mechanisms suppress its biogenesis. Compatible with this scenario, we find that the expression of the *let-7a-1/7f-1/7d* poly-cistronic miRNA precursor is widespread.

One family member, *let-7d*, is unique to this locus and its expression in the brain was abolished by deletion of the CGI on both alleles. A second miRNA, *let-7a-1*, was reduced to 15% of the wildtype levels in the homozygotes, while the abundance of the third, *let-7f*, was unaffected by the deletion. The abundance of the individual miRNA products in other tissues was not tested, but we note that the precursor RNA is widely expressed, most strongly in the testis and embryo. The increased size of the surviving homozygous animals implies hyper-proliferation of one or more tissues, although increased food intake is also a possible cause. Although this was not investigated further, we note that over-expression of *Lin28a*, which inhibits processing of *let-7* miRNAs, also leads to increased size [29]. This study indicated that this effect was due to increased cell proliferation linked to increased glucose utilization and was independent of insulin-like growth factor 2 (IGF2). Previous studies have implicated *let-7* in regulation of the metabolism and lifespan [30]. Interestingly, *let-7* target genes, *DOTL1*, *HMGA2*, and *CDK6*, are associated with taller stature in humans, consistent with the possibility that LIN28B acts through *let-7* to affect height [31]. It is likely that the phenotypes we observe, including reduced survival, are due to depletion of one or both of the two affected miRNAs, though we cannot rigorously rule out that the loss of the CGI per se is deleterious. RNA-Seq does not identify another transcript on the other strand and transcripts from the nearest promoters for flanking annotated genes, ~30 kb (*Zfp169*) and ~50 kb (*Ptpdc1*) distant on either side, are not obviously affected by loss of the CGI.

Our results offer support for the notion that members of the *let-7* family, despite their identical seed sequence, perform non-identical functions. Specifically, we find that the combined absence of *let-7d* and the depletion of *let-7a* leads to a prominent phenotype in mice characterised by increased body size and reduced survival in a high-level screen. While the origin of this phenotypic specificity is not currently unknown, the availability of our mouse model will facilitate molecular approaches that may shed light on its underlying mechanism.

## 4. Materials and Methods

### 4.1. ESC Differentiation

E14 Tg2a embryonic stem cells (ESCs) were grown in Glasgow MEM (G-MEM; Gibco, Dublin, Ireland) supplemented with 15% FBS (Gibco, batch tested), 1% non-essential amino acids (NAA; Gibco), 1% sodium pyruvate (Gibco), 0.1% β-mercaptoethanol (Gibco), and 1000 units/mL LIF (ESGRO, Merck, Watford, UK). Differentiation of ESCs into embryoid bodies (EBs) and further into neuronal cells was carried out using a 4−/4+ retinoic acid (RA) procedure as previously described [32,33]. Briefly, ESCs were seeded into 15 cm bacterial culture dishes at 12.5 × 10^6^ cells/dish in EB medium (same as ESC culture medium, but 10% FBS and no LIF). After 4 days, EB medium + 5 µM all-trans RA (Sigma-Aldrich, Merck, Watford, UK) was used. After 4 more days, EBs were trypsinised and seeded onto culture dishes coated with poly-DL-ornithine (Sigma) and laminin (Roche Pharmaceuticals, Basel, Switzerland) at 1–2 × 10^5^ cells per cm^2^ in N2 medium (DMEM/F-12 + N2-supplement; Invitrogen). From the next day onwards, cells were cultured in a 1:1 mixture of N2 and neurobasal medium (supplemented with B-27; and L-Glutamin; Invitrogen, Carlsbad, CA, USA) for 5 more days.

### 4.2. ESC Targeting and Generation of KO Mice

To delete the CGI of interest (mm9, chr13:48,640,767-48,642,340) in mice, a targeting vector was generated using a combination of bacterial artificial chromosome (BAC) recombineering and Gateway cloning. A 5′ loxP site was inserted upstream of the CGI and replaced 72 bp of endogenous DNA (mm9, chr13:48,642,634-48,642,705). A STOP/selection cassette containing the following elements was inserted, replacing 14 bp of genomic DNA (mm9, chr13:48,640,254-48,640,267) and containing the following elements: Frt–rabbit beta-globin STOP cassette (Broad/oryCun2, chr1:146,236,667-146,237,815)–3× polyA–rox–SV40 pA–human beta-actin promoter driving a neomycin-resistance cassette–SV40 pA–rox–frt–loxP. C57BL/6N ES cells were electroporated with the targeting vector, and positive clones were injected into blastocysts. Chimeras were mated to produce germ-line transmission offspring. Embryos carrying the STOP allele were treated with cell-permeant CRE to delete the floxed sequence, resulting in Cpgi5563^tm1.1(NCC)WCS^ mice with the KO allele in which 2452 bp (mm9, chr13:48,640,254-48,642,705) containing the CGI were deleted. These mice were maintained on a C57BL/6N background for subsequent analysis.

### 4.3. Mice

All mice used in this study were bred and maintained at the University of Edinburgh or at the Sanger Institute animal facility under standard conditions, and procedures were carried out by staff licensed by the UK Home Office and in accordance with the Animal and Scientific Procedures Act 1986. The work was undertaken under following UK Home Office Procedure Project Licences (PPL): 60/4547, valid from 25 July 2013 and approved by the University of Edinburgh Animal Welfare and Ethics Review Board (AWERB), as well as P77453634, valid from 22 November 2016 and approved by the Sanger Institute AWERB.

### 4.4. Phenotypic Analysis of Mice

Phenotypic analysis was performed according to the systematic phenotyping pipeline [14] at the Sanger Institute, with mice placed on a mouse breeder diet (#5012, LabDiet, London, UK). (https://www.mousephenotype.org/impress/procedures/15). A first pass analysis of the phenotyping data was performed using the reference range method as described [14]. This method aims to highlight phenotypes with large effect sizes and results in conservative phenotypic calls while minimizing false positives. To perform this method, accumulated wild-type (WT) data is used to identify and refine the 95% population range for each parameter studied. Mutant data is compared to the relevant reference range and variant phenotypes are determined using a standardized set of rules (normal rule: 4/7 mutants per sex should be outside of the reference range to count as a phenotype; with manual override possible for unusual data patterns. The same data are also presented on the IMPC website (http://www.mousephenotype.org/data/genes/MGI:5670665) where data was analysed by a statistical pipeline based on the R package Phenstat (http://www.mousephenotype.org/data/documentation/doc-method) and [34]). This allows analysis of high-throughput data from categorical and continuous experiments and accounts for batch-to-batch variation, sexual dimorphism, and body weight. This series of formal statistical tests produces both effect sizes and *p*-values for each parameter measured, which are compared to a pre-set significance threshold of *p* < 0.001 to allow for multiple testing. Phenstat generally identifies more phenotypes with smaller effect sizes, compared to the reference range method.

### 4.5. RNA Extraction

Cells were directly harvested into TriReagent (Sigma). Mouse tissues snap frozen in liquid N2 were homogenised in TriReagent using a Polytron homogeniser. All RNA samples were treated with DNase (DNA-free kit, Thermo Fisher, Invitrogen, Carlsbad, CA, USA).

### 4.6. qPCR Analysis of cDNA

For standard reverse transcription, 1 µg of DNase treated RNA per 20 µl reaction was reverse transcribed using the Revert-Aid kit (Thermo Fisher). A control reaction without reverse transcriptase (-RT) was performed alongside.

To detect miRNA, DNase-treated total RNA was reverse transcribed using the miScript II RT kit (Qiagen, Hilden, Germany) using the HiFlex buffer.

All qPCR reactions were carried out using SensiMix SYBR and Fluoroscein Master Mix (Bioline Reagents Limited, London, UK) on a Roche Lightcycler 480 using the following primers: *KY467470:* (a) qOT29-F1: GCATTCCTCTACCTTTCCAGCTTG, qOT29-R1: TGTGCCCTCCCCCACTATAAATTG, (b) qOT29-F4: AATCATCCCCAGGCCTCAGT; qOT29-R4: GTGACCCCTTATTCCTCCAGCA, (c) qOT29-F3: AGGCCTGCAATGTTCCCACT, qOT29-R3: TCCTTTCTGCCTTGGGTTCGT; qMeCP2-Ex3/4-F1: ACCTTGCCTGAAGGTTGGAC, qMeCP2-Ex3/4-R1: GCAATCAATTCTACTTTAGAGCGAAAA, qCypA-F1: TCGAGCTCTGAGCACTGGAG, qCypA-R: CATTATGGCGTGTAAAGTCACCA, qGapdh-Ex5-F: CATGGCCTTCCGTGTTCCTA, qGapdh-Ex5-R: TCATACTTGGCAGGTTTCTCCA, qAirn-F1: ACTTGTACAAACGGGCGGAAC, qAirn-R1: TTTTCCTTGCCTGTGCGAACC, q5S-F1: CTACGGCCATACCACCCT, q5S-R1: GGTATTCCCAGGCGGTCT, q5.8S-F1: CTTAGCGGTGGATCACTCG, q5.8S-R1: AGTGCGTTCGAAGTGTCGAT, q28S-F2: CGGGTAAACGGCGGGAGTAAC, q28S-R2: TAGGTAGGGACAGTGGGAATCTCG, qKcnq1ot1-F1: TTTGCTGTCTGTGTCACTCAGC, qKcnq1ot1-R1: ATTTGGCTCTGGTTGCTGTGG, q45S-pre-rRNA-F: TGTCTGCCCGTATCAGTAACTGTC, q45S-pre-rRNA-R: CCCTGGCCCGAAGAGAACT, qZfp169-F1: ACTATAGCCACCTTGTCTCCCTG, qZfp169-R1: TTCTGCCCCTTGTTCCAGCT, qPtpdc1-F1: GTATGAGAACCCAGCCCGCT, qPtpdc1-R1: CCATGGCCAGGATATTGTCAGTG. For miRNA levels, following primer assays were used: Mm_let-7a_2, Mm_let-7d_1, Mm_let-7f_1, Hs_SNORD_68_11 (all from Qiagen). Samples were run in triplicate and quantification was made after correction of the *C*_T_ values using a standard curve generated by serial dilutions.

### 4.7. 5′ RACE

DNase treated RNA from EBs was prepared for 5′ RACE using the RLM-RACE kit and SuperTaq Plus (Ambion, Invitrogen, Carlsbad, CA, USA). Gene-specific outer primer: 5′-RACE-OT29-O4: CCCCCTTGTCCTTTCATAATTATCCT, gene-specific inner primer: 5′-RACE-OT29-O3: CAGACGATCACTTCTGACACAGACC. PCR fragments were gel purified (Qiaquick PCR purification kit, Qiagen) and ligated into pGEM-T Easy (Promega, Madison, WI, USA). Individual clones were sequenced.

### 4.8. RNA Seq

For RNA Seq in mouse tissues, DNase treated RNA was subject to rRNA depletion using the Ribo-Zero kit (ESCs, EBs, neuronal cells; Epicentre, Middleton, WI, USA) or Ribo-Zero Gold kit (brain; Epicentre), RNA-Seq libraries were prepared using the Script-Seq Library Prep kit (ESCs, EBs, neuronal cells; Epicentre) or Script-Seq V2 Library Prep kit (brain; Epicentre). Size selection was performed using AMPure XP beads (0.7vol beads; Agencourt). RNA-Seq 75b paired end, strand specific reads were sequenced on an Illumina Hi-Seq and subsequently mapped to the mm9 reference transcriptome. Tophat v1.4.1 with Bowtie 2.0.0.6 was used to align reads to transcript sequences defined by the UCSC gene annotations provided by iGenomes [35]. The resulting files were filtered to extract properly paired, primary alignments and separated into forward and reverse strand using Samtools [36]. Bam files were converted to bigWig files for genome wide visualisation using bedtools genomeCoverageBed [37], and the UCSC utility wigToBigWig.

For RNA Seq in human tissues, data for wildtype LUHMES cells were taken from https://doi.org/10.1101/391904. Briefly, LUHMES (human embryonic mesencephalic cells) were differentiated into neurons for 9 days [38,39]. RNA was treated with DNase (DNA-free, Ambion), rRNA depleted (RiboZero Gold kit, Epicentre), and an RNA-Seq library was prepared using a Script-Seq V2 Library Prep kit (Epicentre). RNA-Seq libraries were sequenced as 100 bp pair-end reads on Hi-Seq platforms. All paired-end sequencing reads were trimmed, and quality controlled using Trimmomatic version 0.33 [40]. The filtered reads were then mapped using STAR version 2.4.2 [41] using hg19 human genome assembly and Ensembl 74 release for annotation.

RNA-Seq data have been submitted to Gene Expression Omnibus under accession GSE123951.

### 4.9. Cap-IP

Cap-IP was performed based on [42] with modifications. Anti-m3-G-/m7G-cap (Synaptic Systems, Cat. No. 201 001) antibody or rabbit IgG (Invitrogen 10500C) were coupled to Protein G Dynabeads (ThermoFisher). 10 µg DNase treated RNA was immunprecipitated overnight at 4 °C in RNA-IP buffer (50 mM Tris-HCl, pH 7.4, 150 mM NaCl, 1% Igepal CA-630, 1 mM EDTA pH 8.0, RNasin ribonuclease inhibitor (Promega), cOmplete protease inhibitor (Roche)). Beads were washed 5× in RNA-IP buffer, and RNA was eluted using RNA-elution buffer (50 mM Tris-HCl, pH 7.4, 450 mM NaCl, 0.4% SDS) for 5 min at room temperature. After extraction with acid phenol/chloroform (Ambion) and ethanol precipitation, samples were reverse transcribed and subjected to qPCR as described above.

### 4.10. Nuclear/Cytoplasmic Fractionation

RNA was separated into nuclear and cytoplasmic fractions using a modified protocol from Sambrook and Russell (Molecular Cloning, 3rd edition). Embryoid bodies were washed 3x with ice-cold PBS and resuspended in lysis buffer (0.14 M NaCl, 1.5 mM MgCl2, 10 mM Tris-HCl, pH 8.6, 0.5% Igepal CA-630, 10 mM Vanadyl-Ribonucleoside complex) and homogenised using a Dounce. This was loaded onto a sucrose cushion (lysis buffer containing 24% *w*/*v* sucrose and 1.5% Igepal CA-630). After incubation on ice for 5 min, cells were fractionated by centrifugation at 10,000× *g* for 20 min at 4 °C. The upper turbid layer containing the cytoplasmic fraction was mixed 1:1 with Proteinase K buffer (0.2 M Tris-HCl, pH 7.5, 25 mM EDTA, pH 8.0, 0.3 M NaCl, 2% SDS) and treated using 200 µg/mL Proteinase K for 30 min at 37 °C. The sample was extracted using acid phenol/chloroform (Ambion), ethanol precipitated, and dissolved in nuclease-free water. The nuclear phase was resuspended in TriReagent (Sigma), RNA was extracted according to the manual. Both, the nuclear and cytoplasmic fractions were DNase treated using the DNA-free kit (Ambion); whilst the cytoplasmic fraction was DNase-treated once, the nuclear fraction was DNase treated twice. RNA was reverse transcribed and analysed by qPCR as above.
